# Detection of Antibodies against the Acetylcholine Receptor in Patients with Myasthenia Gravis: A Comparison of Two Enzyme Immunoassays and a Fixed Cell-Based Assay

**DOI:** 10.3390/jcm12144781

**Published:** 2023-07-19

**Authors:** Caterina Maria Gambino, Luisa Agnello, Anna Maria Ciaccio, Concetta Scazzone, Matteo Vidali, Vincenzo Di Stefano, Salvatore Milano, Filippo Brighina, Giuseppina Candore, Bruna Lo Sasso, Marcello Ciaccio

**Affiliations:** 1 Department of Biomedicine, Neurosciences and Advanced Diagnostics, Institute of Clinical Biochemistry, Clinical Molecular Medicine and Clinical Laboratory Medicine, University of Palermo, 90127 Palermo, Italy; caterinamaria.gambino@unipa.it (C.M.G.); luisa.agnello@unipa.it (L.A.); concetta.scazzone@unipa.it (C.S.); giuseppina.candore@unipa.it (G.C.); bruna.losasso@unipa.it (B.L.S.); 2 Department of Laboratory Medicine, University Hospital “P. Giaccone”, 90127 Palermo, Italy; salvatore.milano@unipa.it; 3 Department of Health Promotion, Maternal and Infant Care, Internal Medicine and Medical Specialties “G. D’Alessandro”, University of Palermo, 90127 Palermo, Italy; annamaria.ciaccio@unipa.it; 4 Foundation IRCCS Ca’ Granda Ospedale Maggiore Policlinico, 20122 Milan, Italy; matteo.vidali@gmail.com; 5 Department of Biomedicine, Neurosciences and Advanced Diagnostics, Unit of Neurology, University of Palermo, 90127 Palermo, Italy; vincenzo.distefano@unipa.it (V.D.S.); filippo.brighina@unipa.it (F.B.)

**Keywords:** myasthenia gravis, diagnosis, acetylcholine receptor antibody, CBA, ELISA

## Abstract

The detection of serum anti-acetylcholine receptor (AChR) antibodies is currently an important tool for diagnosing myasthenia gravis (MG) since they are present in about 85% of MG patients. Many serological tests are now available. Nevertheless, results from these tests can be different in some patients. The aim of this study is to compare the sensitivity of a commercially available fixed cell-based assay (F-CBA) to that of enzyme-linked immunosorbent assay (ELISA) kits for anti-AChR detection in patients with a diagnosis of MG. Overall, 143 patients with a confirmed MG diagnosis were included in the study. The detection and measurement of serum anti-AChR antibodies were performed by three analytical methods, namely, a competitive ELISA (cELISA), an indirect ELISA (iELISA), and an F-CBA, according to the manufacturers’ instructions. Anti-AChR antibody titers were positive in 94/143 (66%) using the cELISA, in 75/143 (52%) using the iELISA and in 61/143 (43%) using the F-CBA (adult and/or fetal). Method agreement, evaluated by concordant pairs and Cohen’s kappa, was as follows: cELISA-iELISA: 110/143 (77%), k = 0.53 (95%CI 0.40–0.66); cELISA-F-CBA: 108/143 (76%), k = 0.53 (95%CI 0.41–0.66); iELISA-F-CBA: 121/143 (85%), k = 0.70 (95%CI 0.57–0.80). Our findings show that the cELISA has better analytical performance than the iELISA and F-CBA. However, the iELISA and F-CBA show the highest concordance.

## 1. Introduction

Myasthenia gravis (MG) is an autoimmune neuromuscular disease characterized by autoantibodies targeting proteins in the neuromuscular junction of the skeletal muscles [1].

The clinical manifestations of the disease differ from mild and focal weakness to myasthenic crisis, an acute respiratory paralysis that requires intensive care [2,3,4]. MG symptoms can be limited to the eye muscles, commonly called ocular MG (OMG), or involve other skeletal muscle symptoms, leading to generalized MG (GMG).

Antibody testing is crucial to confirm the clinical suspicion of MG and guide the management of patients [5].

The most common antibody type in the sera of MG patients is against the nicotinic acetylcholine receptor (AChR), which consists of α, β, δ and ε subunits (adult-type AChR-ε) or α, β, δ and γ subunits (fetal-type AChR-γ). Antibodies against all five AChR subunits are detectable in about 85% of patients with GMG and about 50% of patients with OMG [5,6]. They also represent a useful serological biomarker for thymoma, which can be detected in 10–20% of MG patients [7].

About 5–10% of MG patients have antibodies against muscle-specific tyrosine kinase (MuSK) [8,9,10]. Anti-AChR and anti-MuSK are very specific, and, in practice, their detection in patients with suggestive symptoms confirms the diagnosis. When the clinical suspicion of MG arises, anti-AChR antibodies are first tested, followed by Anti-MuSK in AChR-negatives, according to the Italian recommendations for the diagnosis and treatment of myasthenia gravis [5]. Only in a few sporadic cases are both antibodies, i.e., anti-AChR and anti-MuSK, present in the same patient. In recent years, new antibody targets have been identified in seronegative MG patients, including antibodies against lipoprotein-receptor-related protein 4 (LRP4), agrin, collagen, antistriational muscle (Kv1.4, titin and ryanodine receptors) and cortactin [11,12,13,14]. However, despite the progress achieved in serological testing, no antibodies can be detected in around 1–15% of MG patients [15]. This may be related to the low sensitivity of current testing methodologies.

Various analytical methods are available for serological analysis, including the radioimmunoprecipitation assay (RIPA), enzyme-linked immunosorbent assay (ELISA), dot-blot testing and a commercial biochip based on a fixed cell-based assay (F-CBA), which measures antibodies against AChR and MuSK simultaneously [16,17,18,19,20,21,22,23]. F-CBAs and live cell-based assays (L-CBA) are reported to have higher sensitivity compared to RIPAs or ELISAs. Notably, it has been reported that discordant results may be achieved for identical samples tested by different analytical methods.

In this study, we aimed to compare the performance of three analytical methods, namely, a competitive ELISA (cELISA), an indirect ELISA (iELISA), and an F-CBA, in detecting AChR antibodies in patients with MG.

## 2. Materials and Methods

### 2.1. Study Population

We performed an observational retrospective study at the University Hospital “P. Giaccone”, Palermo, Italy, including 143 patients (66 males, 77 females, median age 61 years) with a confirmed diagnosis of MG according to the International Consensus Guidance for Management of MG [24]. We enrolled blood donors as age- and sex-matched healthy controls.

Clinical data, including MG crisis and the state of immunosuppressive treatment, were recorded by reviewing medical records. The presence of thymoma was investigated in all patients by means of computed tomography or magnetic resonance imaging scanning of the mediastinum.

MG patients were classified into five groups according to the Myasthenia Gravis Foundation of America (MGFA) clinical classification at the onset of myasthenic symptoms and at each follow-up [25].

The study was conducted in accordance with the ethical standards as formulated in the Helsinki Declaration and approved by the ‘Palermo I’ Ethical Committee (nr. 05/2021) on 19 May 2021.

For each subject enrolled, we collected blood samples in dry tubes to obtain sera. The latter was separated within 3 h after drawing and stored at −80 °C until analysis. All analyses were performed at the Institute of Clinical Biochemistry, Clinical Molecular Medicine and Clinical Laboratory Medicine, University of Palermo.

### 2.2. Anti-AChR Antibody Assays

Detection and measurement of serum anti-AChR antibodies were performed by three different assays (Table 1).

**Figure 1:**
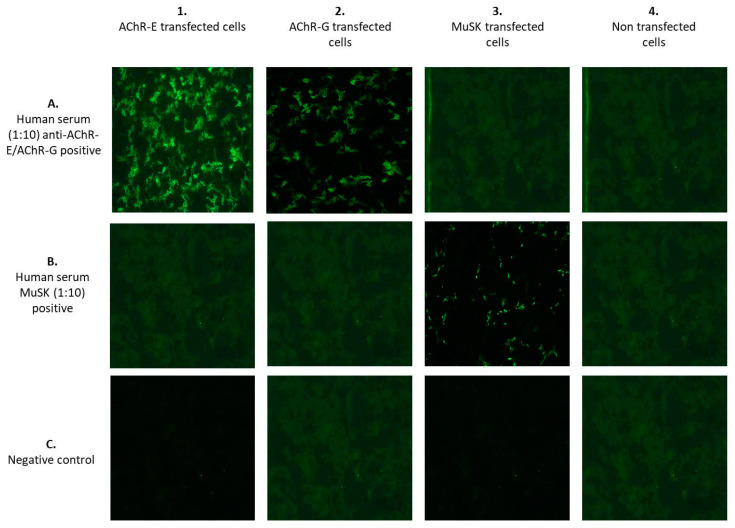
BIOCHIP mosaic for MG [26]. On a standard-sized slide, there are five incubation fields each with four different substrates: (1) Anti-adult acetylcholine receptor (AChR-E) positive transfected cells; (2) Anti-fetal acetylcholine receptor (AChR-G) positive transfected cells; (3) Anti-MuSK positive transfected cells; (4) No transfected cells.

I. cELISA was performed using the commercially available kit RSR AChR Autoantibody (RSR Ltd., Cardiff, UK) according to the manufacturer’s instructions [18]. It is a non-isotopic assay based on the ability of AChR autoantibodies to compete with three different AChR monoclonal antibodies (MAbs 1–3) for binding sites on affinity-purified fetal and adult-type AChR. One MAb (MAb1) is coated onto ELISA plate wells, and the other two are labeled with biotin and used in the assay in the liquid phase. In the absence of serum AChR autoantibodies, a sandwich is formed among MAb1, the AChR and the two biotinylated MAbs, which are subsequently detected by the addition of streptavidin peroxidase, which is bound specifically to biotin. In the presence of serum AChR autoantibodies, the formation of the sandwich fails, and the amount of biotinylated MAbs is reduced. A higher concentration of serum AChR autoantibodies is associated with greater inhibition of MAb-biotin binding. The concentration of AChR autoantibodies is measured in nmol/L, and a raised value above the cut-off (0.5 nmol/L) is considered nearly 100% specific for MG.
II. iELISA was performed using the commercially available Anti-Acetylcholine Receptor ELISA (IgG) kit from Euroimmun (Lübeck, Germany) according to the manufacturer’s instructions. The stabilized antigen is coated onto the surface of the microwells to serve as antigenic substrates. The manufacturer-recommended cut-off values were used as follows: <0.4 nmol/L, negative; 0.4–0.5 nmol/L, borderline; >0.5 nmol/L, positive.
III. F-CBA was performed using the commercially available kit MG Mosaics (Euroimmun, Lübeck, Germany) based on the principle of BIOCHIP, which simultaneously detects different antibodies. It is performed by transfecting the fixed HEK cells with complementary DNA expressing human AChR α, β, δ and ε/γ subunits and rapsyn-enhanced green fluorescent protein. The transfected cells are incubated with serum samples diluted with phosphate-buffered saline containing 0.002% Tween 20 in 1:10 dilutions for 30 min at room temperature. Measurement of antibody binding is performed by indirect immunofluorescence. In the second and third steps, the linked antibodies are stained with biotin-labeled anti-human IgG, followed by fluorescein isothiocyanate-labeled avidin and made visible with the fluorescence microscope. A smooth or fine-to-granular green fluorescence signal is detected both in the cytoplasm and at the cell surface membrane. The BIOCHIP slide is composed of combinations of 4 substrates for each patient’s test: (1) recombinant cells transfected with AChR-ε; (2) recombinant cells transfected with AChR-γ; (3) recombinant cells transfected with MuSK; and (4) untransfected recombinant cells used as negative controls (Figure 1). The fluorescence was scored by a DMIRE2 Leica fluorescence microscope (Leica, Milan, Italy) with a 20× lens. Pictures were acquired by a digital camera model DC250 Leica, using the acquisition software Qfluor550 Leica (V7.7.1). Two expert operators, who worked independently and were blinded to the clinical data, interpreted the results. Unclear results were repeated until consensus was achieved.

Table 1 describes detailed performance characteristics of the three different assays.

### 2.3. Statistical Analysis

Statistical analysis was performed by R Language v.4.2.1 (R Foundation for Statistical Computing, Vienna, Austria), with additional packages including “dplyr”, “ggplot2”, “boot”, “VCA” and “irr”. Method comparison, using continuous values, was evaluated by non-parametric Passing–Bablok regression. Concordance was also evaluated as a percentage of concordant pairs and by Cohen’s kappa with its 95% confidence interval. Analytical performances were evaluated by calculating sensitivity, specificity, positive predictive value and negative predictive value and by ROC curve analysis. AUCs were compared by the DeLong method.

## 3. Results

**Figure 2:**
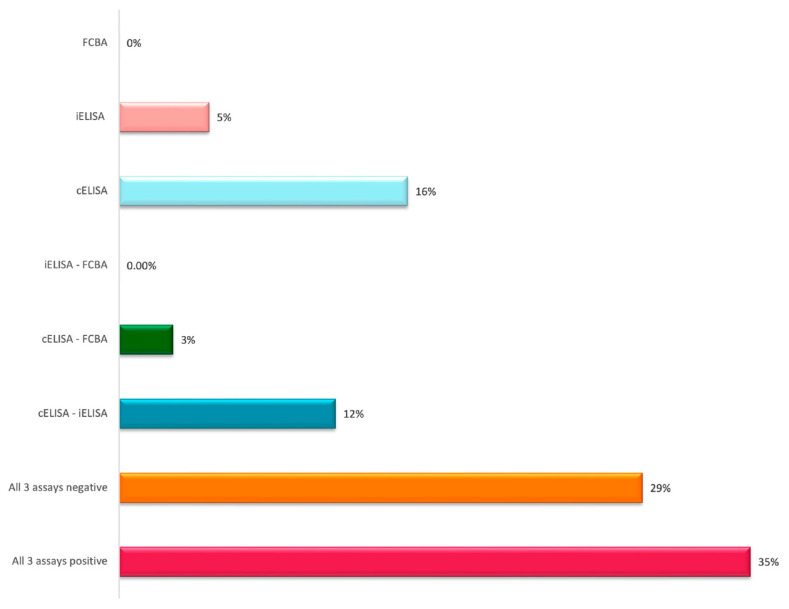
Combined data of the three assay measurements.

AChR antibodies were measured in 143 MG patients (M:F 66:77, median age 61 years) and seventy healthy controls (M:F 28:32, median age 50 years). Table 2 shows the demographic and clinical characteristics of MG patients. Anti-AChR antibody titers were positive in 94/143 (66%) using the cELISA, in 75/143 (52%) using the iELISA and in 61/143 (43%) using the F-CBA (adult and/or fetal) (Table 3, Table 4 and Table 5). Figure 2 shows the combined data of positivity for all three assays evaluated.

The method agreement, evaluated by concordant pairs and Cohen’s kappa, was as follows: cELISA-iELISA: 110/143 (77%), k = 0.53 (95%CI 0.40–0.66); cELISA-F-CBA: 108/143 (76%), k = 0.53 (95%CI 0.41–0.66); iELISA-F-CBA: 121/143 (85%), k = 0.70 (95%CI 0.57–0.80). In the comparison of cELISA-iELISA, 26 subjects were positive for cELISA but negative for iELISA. Only 3 out of these 26 subjects resulted in being positive according to the F-CBA (1 adult only, 2 both adult and fetal). Among seven patients negative for cELISA but positive for iELISA, none was positive according to the F-CBA.

**Figure 3:**
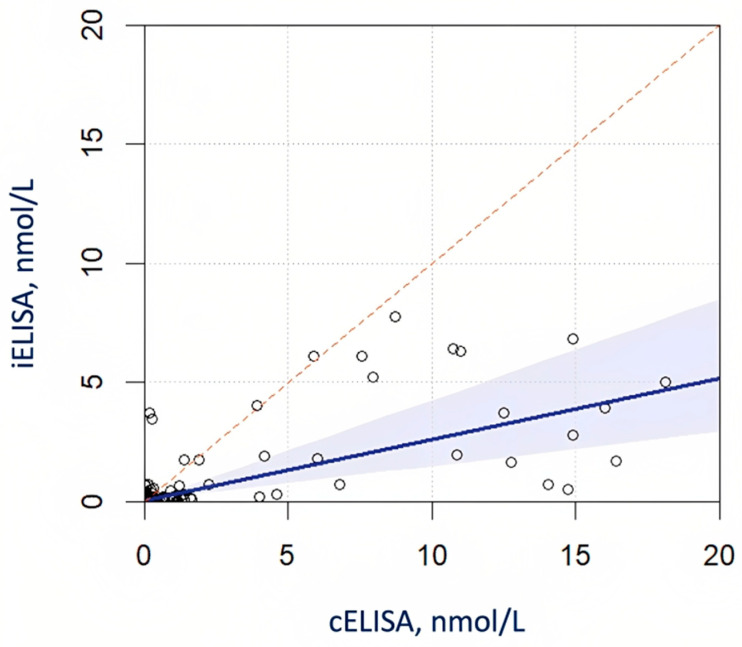
Passing–Bablok regression for subjects with cELISA < 20 mmol/L and iELISA < 8 mmol/L.

The quantitative anti-AChR antibody results, measured by cELISA and iELISA, were further compared. To this aim, all of the subjects’ results that were significantly above the detection range, i.e., >20 nmol/L for cELISA or >8 nmol/L for iELISA, were excluded, resulting in 99 valid anti-AChR antibody pairs. From Passing–Bablok regression analysis, we obtained a slope and intercept, respectively, equal to 0.26 (95%CI 0.14 to 0.42) and 0.03 (−0.02 to 0.06) (Figure 3). Out of 44 subjects excluded from the regression, 27 had both cELISA > 20 nmol/L and iELISA > 8 nmol/L, 7 displayed cELISA > 20 nmol/L but iELISA < 8 nmol/L, and up to 10 had cELISA < 20 nmol/L but iELISA > 8 nmol/L.

The healthy controls received negative results according to all three AChRAb tests. Sensitivity (Se), specificity (Sp), positive predictive value (PPV) and negative predictive value (NPV) for all AChRAb tests, and their different combinations, are reported in Table 6.

**Figure 4:**
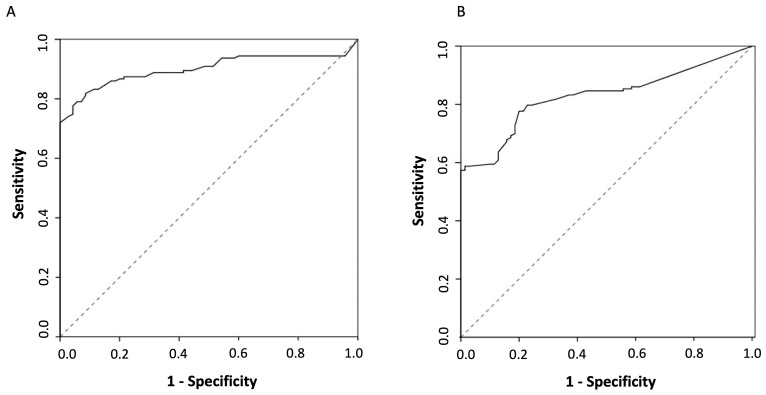
(**A**) ROC curve of the cELISA test for MG detection. (**B**) ROC curve of the iELISA test for MG detection.

ROC curves for the cELISA and iELISA were reported in Figure 3. AUCs were 0.900 (95%CI 0.857–0.942) for cELISA and 0.828 (95%CI 0.774–0.882) for iELISA (shown in Figure 4A,B, respectively). The difference between the AUCs was statistically significant (difference: 0.072, 95%CI 0.009–0.135; Delong *p* = 0.0261).

## 4. Discussion

The detection of anti-AChR antibodies is currently an important tool for diagnosing MG since even very low titers of serum anti-AChR antibodies are sufficient to confirm the clinical suspicion [9]. Moreover, the gradually increasing titers of anti-AChR antibodies may be detected up to 2 years before the onset of typical MG symptoms [27].

RIPAs represent the gold standard for detecting anti-AChR antibodies due to their high specificity and sensitivity, reaching 99% and 85%, respectively [13]. Additionally, a RIPA is also a quantitative method, and, thus, the quantification of antibody levels could be helpful for patient monitoring. However, RIPAs have some limitations, mainly being the use of radioactive reagents. Over time, non-radioactive alternatives have been developed and commercialized. Among these, ELISAs are the most used. In the last decade, CBAs have also been introduced in MG diagnosis. CBA is a methodology based on the expression of high levels of antigen, i.e., AChR, in the membrane of cells, which can be live (L-CBA) or fixed (F-CBA). L-CBAs have been proven to be highly specific and sensitive, even more than RIPAs [28]. However, the use of L-CBAs in clinical practice is hampered because it requires expertise and cell-culture facilities. The limitations of RIPAs and L-CBAs can be overcome by using an FCBA. Indeed, the latter is not based on radioactive material and is less technically demanding and time-consuming than an L-CBA. Thus, similarly to an ELISA, it could be easily implemented in clinical practice.

In this study, we first compared the analytical performance of an F-CBA with two ELISA assays in a population of patients with MG. The main findings of our study can be summarized as follows: (i) the cELISA detected the most AChR antibodies in comparison to both the iELISA and F-CBA; (ii) the iELISA and F-CBA had the highest concordance; (iii) the comparison of continuous titers between the iELISA and cELISA did not provide additional information given the different magnitude and the extension of the results (different limit of the analytical range). Indeed, a slope of 26% indicated that one method overall gave values that are one-third of another; (iv) the cELISA showed the highest analytical performances. Se and NPV increased by adding the iELISA (combined double strategy of cELISA+iELISA), respectively, from 66% to 71% and from 59% to 63%. No further increase was observed by adding iIF (combined triple strategy). Overall, our findings show that the cELISA has better analytical performance than the iELISA and F-CBA. An ELISA has some advantages over CBAs, both live and fixed. First, a CBA undergoes subjective interpretation, which is related to the expertise of the reader and could undermine its reproducibility. Additionally, a CBA provides only qualitative results. Finally, a cELISA does not require dilution.

To date, only a few studies have evaluated the performance of F-CBAs for anti-AChR detection. We previously demonstrated that the F-CBA for MG diagnosis did not accurately identify low anti-AChR and anti-MuSK levels, which were detected by an ELISA [26]. Mirian et al. showed that F-CBAs have similar specificity and higher sensitivity compared to RIPAs but lower performance than L-CBAs [22]. Also, Spagni et al. found that L-CBAs are more sensitive than F-CBAs [29].

Overall, the literature comparing live and fixed CBAs for detecting neural antibodies shows a decreased sensitivity for F-CBAs [30].

In our study, we compared three analytical methods that are commercially available and easy to introduce in clinical laboratories.

Notably, in our study, we found a percentage of seronegative MG patients, in accordance with the literature [31]. The seronegativity could result from low affinity or low levels of antibodies requiring more sensitive assays. Thus, the implementation of analytical methods with high sensitivity and specificity and that are easy to perform are still sought after.

The main limitation of our study is the lack of a comparison with a RIPA.

Further studies on larger cohorts comparing the diagnostic performance of commercially available assays for anti-AChR antibody detection are required.

## Figures and Tables

**Table 1:** Characteristics of competitive ELISA, sandwich ELISA, and F-CBA compared in this study.

Assays	Competitive ELISA	Sandwich ELISA	F-CBA
Status	CE-IVD	CE-IVD	CE-IVD
Antibody isotype	IgG	IgG	IgG
Test format	96-well microplate	96-well microplate	10 × 5 slides
Sample type	Serum	Serum and plasma	Serum and plasma
Sample dilution	Non-dilution	1:26	1:10
Conjugate	Streptavidin-HRP	HRP-rabbit anti-human IgG	Biotin-labeled anti-human IgG, FITC-labeled avidin
Incubation time (hours)	24	3	2
No calibrators	4	5	NA
Calibration range	0.5–20 nmol/L (0.5, 1, 6.5, 20)	0–8 nmol/L (0, 0.25, 0.75, 2.5, 8)	NA
Cut-off value	Negative: <0.45 nmol/L Positive: ≥0.45 nmol/L	Negative: <0.40 nmol/L Borderline: ≥0.40 < 0.50 nmol/L Positive: ≥0.50 nmol/L	No reaction at 1:10 Positive reaction at 1:10
Limit of detection	0.25 nmol/L	0.11 nmol/L	NA

ELISA, Enzyme-linked immunoassay; F-CBA, fixed cell-based assay; CE-IVD, CE-marked in vitro diagnostic medical device; HRP, horseradish peroxidase; FITC, fluorescein isothiocyanate; NA, not available.

**Table 2:** Demographic and clinical characteristics of MG patients.

Variable	Descriptive Statistics
**Demographic**	
N	143
Sex, M (%)	46%
Age, years	61
**Clinical**	
Age at onset, years	52 (41–62)
Type, generalized:ocular	67%:33%
MGFA at onset	
I	29%
II	48%
III	16%
IV	6%
V	1%
MGFA at follow-up	
I	31%
II	47%
III	21%
IV	1%
V	0%
Thymoma	18%
Thymic hyperplasia	12%
Thyreopathy	23%
Autoimmune disease	21%
Kidney disease	8%
Neuropathy	14%
Hypertension	38%
Cardiovascular disease	15%
Osteoporosis	26%
Eye disease	12%
Gastrointestinal disease	15%
Diabetes	12%
Hematological disease	8%
Cancer disease	7%
Psychiatric disorder	14%
Respiratory disease	11%
Neurological comorbidities	22%
Pyridostigmine	74%
Prednisone	73%

**Table 3:** Cross-table for cELISA vs. iELISA positivity.

Method	iELISA Pos	iELISA Neg	Total
cELISA pos	68	26	94
cELISA neg	7	42	49
Total	75	68	143

**Table 4:** Cross-table for cELISA vs. IFA CBA positivity.

Method	IFA CBA Pos	IFA CBA Neg	Total
cELISA pos	60	34	94
cELISA neg	1	48	49
Total	61	82	143

**Table 5:** Cross-table for iELISA vs. IFA CBA positivity.

Method	IFA CBA Pos	IFA CBA Neg	Total
iELISA pos	57	18	75
iELISA neg	4	64	68
Total	61	82	143

**Table 6:** Analytical performances of the tests and their combinations. PPV: Positive predictive value; NPV: negative predictive value.

Analytical Method	Sensitivity	Specificity	PPV	NPV
cELISA	66%	100%	100%	59%
iELISA	52%	100%	100%	51%
F-CBA	43%	100%	100%	46%
cELISA + iELISA	71%	100%	100%	63%
cELISA + F-CBA	66%	100%	100%	59%
iELISA + F-CBA	55%	100%	100%	52%
cELISA + iELISA+ F-CBA	71%	100%	100%	63%

## Data Availability

The data that support the findings of this study are available from the corresponding author, upon reasonable request.
